# A USV-UAV Cooperative Trajectory Planning Algorithm with Hull Dynamic Constraints

**DOI:** 10.3390/s23041845

**Published:** 2023-02-07

**Authors:** Tao Huang, Zhe Chen, Wang Gao, Zhenfeng Xue, Yong Liu

**Affiliations:** 1 Institute of Cyber-Systems and Control, Zhejiang University, Hangzhou 310027, China; 2 Intelligent Perception and Control Center, Huzhou Institute of Zhejiang University, Huzhou 313098, China; 3 Science and Technology on Complex System Control and Intelligent Agent Cooperation Laboratory, Beijing 100191, China

**Keywords:** USV-UAV cooperation, trajectory generation, under-actuated constraint, numerical optimization, hull dynamics

## Abstract

Efficient trajectory generation in complex dynamic environments remains an open problem in the operation of an unmanned surface vehicle (USV). The perception of a USV is usually interfered by the swing of the hull and the ambient weather, making it challenging to plan optimal USV trajectories. In this paper, a cooperative trajectory planning algorithm for a coupled USV-UAV system is proposed to ensure that a USV can execute a safe and smooth path as it autonomously advances through multi-obstacle maps. Specifically, the unmanned aerial vehicle (UAV) plays the role of a flight sensor, providing real-time global map and obstacle information with a lightweight semantic segmentation network and 3D projection transformation. An initial obstacle avoidance trajectory is generated by a graph-based search method. Concerning the unique under-actuated kinematic characteristics of the USV, a numerical optimization method based on hull dynamic constraints is introduced to make the trajectory easier to be tracked for motion control. Finally, a motion control method based on NMPC with the lowest energy consumption constraint during execution is proposed. Experimental results verify the effectiveness of the whole system, and the generated trajectory is locally optimal for USV with considerable tracking accuracy.

## 1. Introduction

Unmanned surface vehicles (USVs) are a kind of specific ships with the ability of autonomous mission execution, which are widely used in various applications, including marine resource exploration, water resource transportation, patrol and defense in key areas and river regulation [1,2]. Progress has been made in a large number of research areas, including environmental perception [3,4], formation control [5,6], navigation [7,8], and so on. Environmental perception and trajectory generation are the two most important techniques when the USVs are executing in unknown environments. In particular, when the environment contains dynamic obstacles, USVs struggle to achieve accurate trajectory planning and tracking due to the lack of effective obstacle information. As a result, the autonomous navigation system may fail.

During the navigation process of a USV, the sensing devices, such as radar or camera, are located at a low observation point, which is detrimental to environmental perception because the adjacent obstacles in the front and behind will block each other. Moreover, the input of the sensors often contains noise caused by hull shaking on the water. This makes precise environmental perception a difficult problem for USVs and affects the success rate of trajectory generation. Usually, simultaneous localization and mapping (SLAM) [9] technology is required to construct the global map. However, this kind of method requires a huge computational load, and it is intractable to deal with dynamic objects in the water environment.

**Figure 1:**
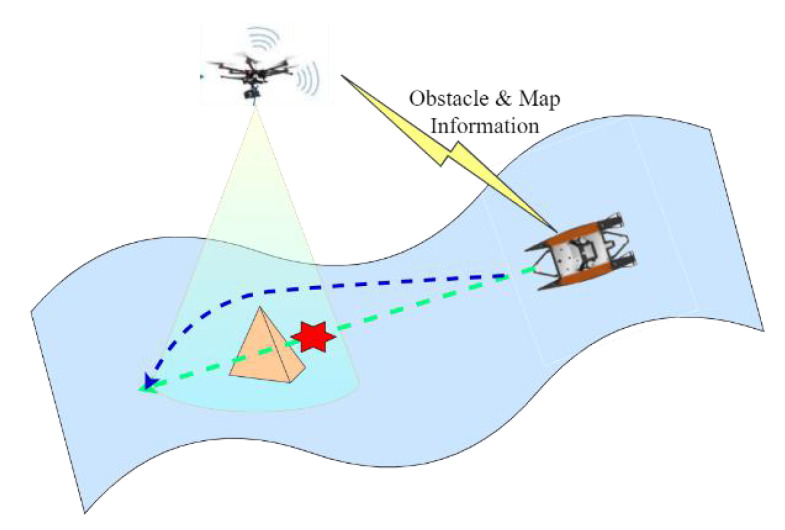
An illustration of the USV-UAV cooperative system, where the UAV provides wide obstacles and map information to guide the USV to generate an obstacle avoidance trajectory.

A feasible solution is to design a USV-UAV cooperative system to tackle the above problems, where the unmanned aerial vehicle (UAV) plays the role as a flying sensor. As shown in Figure 1, the USV has long cruise capability, but its perception is disturbed and limited by the circumstance. Hence the UAV flies over the USV, providing more stable and comprehensive information. Semantic segmentation [10,11] and 3D projection are used in this paper to transfer obstacle information in the field of vision of the UAV to the coordinate system of the USV. Semantic segmentation extracts pixel information of environmental obstacles, and a camera projection model helps to transfer the pixel information to 3D information. By doing this, global map information around the USV can be obtained efficiently and in real-time, implying the USV-UAV cooperative system can improve the perception ability of the USV effectively, allowing the USV to perform tasks in more complex water circumstances.

An initial obstacle avoidance trajectory is firstly generated by a graph-based search method [12]. However, such a method was originally designed for path searching on vast geographical scenarios, which does not consider the USV’s dynamic characteristics. On the other hand, USV is famous for its under-actuated motion characteristics [13], which makes it hard to be controlled well, even when an optimal trajectory is planned. In this paper, we design a numerical optimization method to optimize the trajectory. Specifically, we take the hull dynamic constraints into account when modeling the optimization problem. As a result, the generated trajectory not only allows the obstacle avoidance rule, but also fits the motion characteristics of a USV. This makes the generated trajectory easier to be tracked under the same control conditions.

Finally, a control method with the lowest energy consumption per execution task is designed under a new numerical optimization problem. It ensures that the power consumption is optimal when the USV is actuated to track the given optimal trajectory, which is a very useful technique in real-world applications. The performance of the trajectory generation and tracking is comprehensively compared and analyzed in the simulated environments, and it verifies the effectiveness of our proposed novel framework.

In summary, the contributions of this paper are listed as follows.

- A novel USV-UAV cooperative system is proposed, where the UAV acts as a flying sensor to provide global map information around the USV by semantic segmentation and 3D projection, providing more comprehensive and effective perception results for navigation planning.
- A numerical optimization problem is formulated during the trajectory generation process. It considers the hull under-actuated dynamic constraints and perception of the UAV, which can generate a fuel-saving trajectory in real-time optimization.
- The lowest energy consumption control law is proposed to track the generated trajectory efficiently and accurately, and extensive experiments are conducted to verify the effectiveness of the USV-UAV cooperative system.

## 2. Related Works

### 2.1. Trajectory Planning for USV

Trajectory planning aims to automatically generate an obstacle avoidance trajectory for a USV when the local or global map is given. Among existing methods, the mainstream trajectory planning methods are mainly divided into two categories, i.e., path search and trajectory generation.

There are two research directions for the path search methods, including graph search and random sampling. Typical graph search methods include the A* [14] and Dijkstra [15] algorithm, as well as their derivatives [16]. These methods mainly discretize the known map into interconnected grids and find the shortest path according to the heuristic parameters. The disadvantage of this kind of method is that the search dimension in the large map is expanding, and the calculation time shows a rapid upward trend. Among random sampling methods, typical varieties include RRT [17] and its derivatives [18], which dynamically find feasible paths by randomly sampling points in the map and constructing random exploratory trees. The method can show better performance for large maps, but its shortcomings are also very obvious. It is easy to be guided to a locally optimal solution, and it is difficult to generate feasible paths in narrow areas when system’s computing resources are limited. The common problem of the above methods is that the generated path curvature is discontinuous, and trajectory smoothing is needed  afterward.

For the trajectory generation methods, curve interpolation methods, such as B-spline [19], are commonly used to smooth the trajectory. The smoothness of the trajectory and motion state is guaranteed by the continuity theorem of higher-order derivatives of a curve. Meanwhile, numerical optimization methods are also widely used, such as minimum snap [20] and near-optimal control [21].

Some methods can also combine path search with trajectory generation, such as domain reduction-based RRT* [22] and Hybrid A* [23]. In this paper, the proposed method belongs to the numerical optimization method. It adds the dynamic and kinematic constraints of unmanned craft in the trajectory generation part so that the generated trajectory is more in line with the dynamic characteristics of the hull.

### 2.2. The USV-UAV Cooperative System

With the rapid development of automation and artificial intelligence technology, unmanned aerial vehicle (UAV) technology has made significant progress in recent years. Compared with USV, the advantage of UAV is that it has a broader field of vision and faster movement speed and can provide more comprehensive and effective data information for USV. In addition, UAV has the advantages of flying height and that its communication ability is less affected by the environment. It can be used to provide communication relay services for multiple USVs located in different positions. Due to the strong complementarity between USV and UAV in perception, communication, operation time, and other aspects, researchers have focused on the coordination of having UAV serve USV and have successfully verified that this method can effectively solve the problem mentioned above of self-awareness of a USV. Ref. [24] focused on the search and rescue of USVs in flood scenes and proposed a collaborative mode of manipulating a UAV to establish the global map first, providing complete map information and target localization for subsequent USV planning. Ref. [25] proposed a cooperative formation control algorithm for a single USV and multiple UAVs. The method is based on the leader-follower distributed consensus model, and the position and orientation of each boat are determined by the RGB image color-space features acquired by the UAV camera. Ref. [26] considered the strong search capability of the UAV in the air, combined with the actual target strike capability of the USV, and proposed a two-stage cooperative path planning algorithm on the water and underwater based on the particle swarm optimization algorithm. Ref. [27] proposed an effective game incentive mechanism for the task assignment problem in the cooperative operation of USVs and UAVs, which reduced the task cost and improved the task efficiency. Ref. [28] proposed that the LVS-LVA framework to be applied the cooperative motion control of USV-UAV.

Although, most of these methods are cooperative ways to provide UAV environmental data and perceptual information for the navigation task of a USV. With the development of computer vision technology, the accuracy and robustness of the perception algorithm they use need to be improved. In addition, they did not consider the trajectory of the USV and its tracking control link, and the proposed collaborative framework can not be fully applied to the autonomous navigation task of USVs.

## 3. Cooperative Trajectory Generation

In the USV-UAV cooperative system, the USV has a stable environmental self-supporting ability, and the UAV is flexible and environmentally adaptable. In the process of autonomous navigation of the USV, relying on the wide field of vision and strong environmental perception provided by the UAV, it can generate a more reasonable trajectory and skillfully avoid various kinds of obstacles.

### 3.1. Environmental Perception and 3D Projection

**Figure 2:**
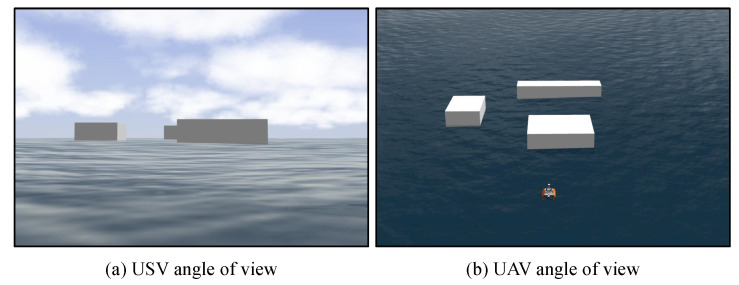
Perspective difference between USV and UAV.

Environmental perception is vital when the USV is performing in unknown water areas. Different observation angles have a significant influence on the observed results. As shown in Figure 2, the USV and UAV have different angles of view. The USV observes the environment from a horizontal perspective, which may lead to serious visual occlusion, whereas the UAV performs environmental perception from a top-down perspective, which enables more accurate map-view information.

Concerning the accuracy of obstacle recognition and the calculation efficiency, we use semantic segmentation technology [29,30] based on deep learning to extract pixel-level obstacle information from the image data obtained by the UAV’s camera. For a given image, the position, shape and size of the obstacles in the environment can be judged by assigning each pixel with a two-categorical label: ‘0’ indicates a safety area and ‘1’ denotes an area in which the obstacles are located.

**Figure 3:**
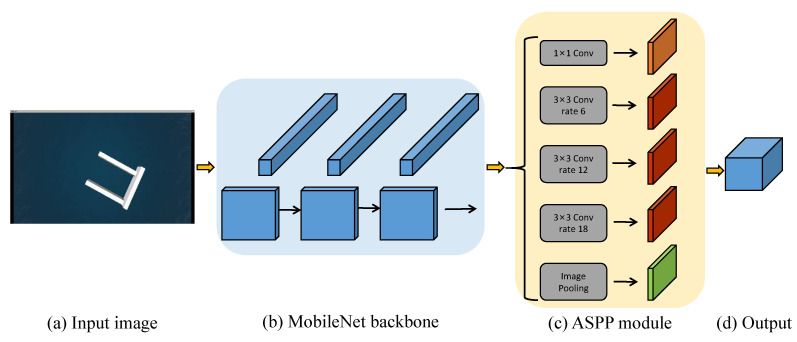
The network architecture of the semantic segmentation algorithm deployed on the UAV.

In this paper, we use DeepLab [10] as the semantic segmentation network and replace the backbone with MobileNet [31]. On the one hand, it reduces the amount of computation. On the other hand, in the process of feature extraction, with the help of the atrous spatial pyramid pooling (ASPP) module, it can effectively improve the global receptive field and the recognition effect. The overall network architecture is illustrated in Figure 3.

After obtaining the pixel coordinates of obstacles in the image, it needs to convert the obstacle coordinate information into a unified global coordinate. We define the coordinate system of the UAV as *U*, the camera coordinate system as *C*, and the global coordinate system as *G*. Thus the transformation from *U* to *C* can be represented by $T_{UC}=\left[R|T\right]\in R^{4\times 4}$, where *R* is the rotation matrix and *T* is the translation matrix. $T_{GU}\cdot T_{UC}$ denotes the transformation matrix from *G* to *C*. Assuming that the coordinates of the obstacle point *m* in the pixel coordinate system are (u,v), according to the imaging principle of the pinhole camera model, the relationship between its position in the camera coordinate system can be expressed as

(1) $$\left\{\begin{array}{cc} u & =f_{x}\cdot \frac{x}{z}+c_{x}\\ v & =f_{y}\cdot \frac{y}{z}+c_{y}, \end{array}\right.$$

where $f_{x}$ and $f_{y}$ denote the focal length in the *x* and *y* direction and $c_{x}$ and $c_{y}$ are the positions of the origin of the image plane, which can usually be regarded as the center of the image. Thus, the relationship between the 3D points in the global coordinate system M=(x,y,z) and the pixel coordinate system m=(u,v) is denoted by

(2) $$s\cdot \left[\begin{array}{c} u\\ v\\ 1\\ 1 \end{array}\right]=\left[\begin{array}{ccc} f_{x} & 0 & c_{x}\\ 0 & f_{y} & c_{y}\\ 0 & 0 & 1\\ 0 & 0 & s \end{array}\right]\cdot T_{GU}\cdot T_{UC}\cdot \left[\begin{array}{c} x\\ y\\ z\\ 1 \end{array}\right],$$

where *s* is the scaling factor, which can be regarded as the depth information of each pixel. In this paper, a binocular camera carried by the UAV is used to obtain the pixel depth *s*. Through this way of 3D coordinate projection, the pixel information sensed by the UAV in real-time can be projected into the global coordinate system, forming the 3D perception ability of the USV to the environment.

### 3.2. Initial Trajectory Generation

In order to generate an obstacle avoidance trajectory, this paper applies the Hybrid A* algorithm [23] to provide an initial path, as shown in Algorithm 1. Given the initial state of the USV ($s=(x_{0},y_{0},\varphi_{0})$) and the navigation target state ($e=(x_{f},y_{f},\varphi_{f})$), the algorithm first puts the initial state into the open list. Then it iteratively reads the node with the lowest cost in the open list as the current parent node, and generates the next child node according to the current node state, system motion mode and obstacle map. Unlike the A* algorithm, the Hybrid A* algorithm adds the orientation dimension to the coordinate system. Therefore, the criteria for reaching the target state is that the distance between the coordinates of the node and the target point is less than the threshold of the reaching distance, and the collision-free Reeds–Shepp curve can be generated through the node state and the target point state.
**Algorithm 1** Trajectory Search with Hybrid A***Input:**$x_{0}$, $x_{f}$, map**Output:** Trajectory *T*1:Function Search($x_{0}$, $x_{f}$, map)2:open ← ϕ, close ← ϕ3:open.push($x_{0}$)4:**while** open is not ϕ **do**5:    $x_{n}$ ← open.pop()6:    close.push($x_{n}$)7:    **if** $x_{n}$.near($x_{f}$) **then**8:        **if** reedsheep($x_{n}$, $x_{f}$) **then**9:           **return** path($x_{f}$)10:  **else**11:      **for** $x_{succ}$∈ successor($x_{n}$) **do**12:           **if** $x_{succ}$.safe() and not exist($x_{n}$, close) **then**13:               *g* ← *g*($x_{n}$) + *g*($x_{succ}$, $x_{n}$)14:               **if** not exist($x_{succ}$, open) or *g* < *g*($x_{succ}$) **then**15:                   pred($x_{succ}$) ←$x_{n}$16:                   h($x_{succ}$) ← Heuristic($x_{succ}$, $x_{f}$)17:                   **if** not exist($x_{succ}$, open) **then**18:                       open.push($x_{succ}$)19:                   **else**20:                       open.rewrite($x_{succ}$)21:**return** null

## 4. Trajectory Optimization and Tracking

The USV is an under-actuated robot operation system where the number of control variables of the system is less than the degrees of freedom of the system. In the trajectory optimization process, if the dynamic constraints of this under-actuated characteristic are added to the optimization process, an optimal trajectory more in line with the characteristics of ship motion can be generated.

### 4.1. Trajectory Optimization with Dynamics

The motion model of the USV is a mathematical model with 6 degrees of freedom when it is complete. For simplicity, we can ignore the motion of the hull in the heave, roll and pitch directions, and simplify it into a 3-degrees of freedom with surge, sway and yaw, represented by *x*, *y* and φ. The mathematical expression of the hull dynamics can be expressed as

(3) $$\left\{\begin{array}{cc} \dot{\eta } & =J(\eta )\nu \\ M\dot{\nu } & =\tau -C(\nu )\nu -D\nu , \end{array}\right.$$

where η=(x,y,φ)$\;\in \;R^{3\times 1}$ denotes the state variables, and ν=(u,v,r)$\;\in \;R^{3\times 1}$ denotes the speed variables. J$\;\in \;R^{3\times 3}$ is the transition matrix, and C$\;\in R^{3\times 3}$ is the Coriolis centripetal force matrix. M$\;\in R^{3\times 3}$ is the inertial matrix, and D$\;\in R^{3\times 3}$ is the damping matrix. $\tau =(\tau_{u},0,\tau_{r})$$\;\in R^{3\times 1}$ is the thrust matrix. For a catamaran, the thrust matrix can be expressed as

(4) $$\left\{\begin{array}{cc} \tau_{u} & =T_{1}+T_{2}\\ \tau_{r} & =(T_{1}-T_{2})\cdot B, \end{array}\right.$$

where $T_{1}$ and $T_{2}$ are the thrusts of two propellers, and *B* is their distance. The USV can be viewed as a linear time-invariant (LTI) system. Its state variables X and control variable τ can be represented by

(5) $$\left\{\begin{array}{cc} X & ={\left[x,y,\varphi ,u,v,r\right]}^{T}\\ \tau & ={\left[\tau_{u},0,\tau_{r}\right]}^{T}. \end{array}\right.$$

The system dynamics are as follows

(6) $$\left\{\begin{array}{c} \;\;\dot{x}=ucos(\varphi )-vsin(\varphi )\\ \;\;\dot{y}=usin(\varphi )+vcos(\varphi )\\ \;\;\dot{\varphi }=r\\ m_{11}\dot{u}-m_{22}ur+d_{11}u=\tau_{u}\\ m_{22}\dot{v}-m_{11}ur+d_{22}v=0\\ m_{33}\dot{r}+\left(m_{22}-m_{11}\right)uv+d_{33}r=\tau_{r}. \end{array}\right.$$

Based on Hybrid A*, the global trajectory is optimized twice with the following constraints, including position, velocity, angular velocity and control input, as well as waypoint state constraints. The reference waypoint state is the sub-optimal trajectory obtained by considering the vehicle model, which can only provide the simulated optimal information of obstacle avoidance, heading speed and other controls. In this paper, we consider the state vector error in the optimization objective function as a soft constraint. The final optimization objective can be represented as

(7) $$min\;\frac{1}{2}\left\{\sum_{i=0}^{N}\left[{\left(X_{i}-X_{i}^{ref}\right)}^{T}W_{x}\left(X_{i}-X_{i}^{ref}\right)+\tau_{i}^{T}W_{\tau }\tau_{i}\right]+\sum_{i=1}^{N}{\left(\tau_{i}-\tau_{i-1}\right)}^{T}W_{u}\left(\tau_{i}-\tau_{i-1}\right)\right\},$$

where $X_{i}^{ref}$ denotes the reference state variables generated by Hybrid A*, and $W_{x}$=diag{50,50,20,15,15,15}, $W_{\tau }$=diag{5,0,5} and $W_{u}$=diag{3,0,3} represent the positive definite, cost and weight matrices, respectively. Moreover, to ensure adequate accuracy in the trajectory, we choose 0.05 s as the sampling period.

We adopt the methods of minimizing the control quantity and minimizing the continuous control difference to ensure that the global trajectory generated by optimization can take into account the trajectory index factors, such as the smoothing of the control quantity and the minimization of the energy consumption at the same time. The overall algorithm flow is shown in Algorithm 2.
**Algorithm 2** Global Trajectory Optimization**Input:**$X_{0}$, $X_{f}$, path**Output:***X*1:Function OptiTraj($X_{0}$, $X_{f}$, path)2:**for***i* = 0 to N **do**3:    **if** *i* == 1 **then**4:        X(i) = $X_{0}$5:    **else if** *i* == N **then**6:        X(i) = $X_{f}$7:    **else**8:        X(i).*x* = $path_{i}$.*x*9:        X(i).*y* = $path_{i}$.*y*10:        X(i).φ = $path_{i}$.φ11:Set constraints *C*12:Set Objective Function *J*13:Optimize(*J*, path, *C*, *X*)14:**return***X*

### 4.2. Tracking Control with NMPC

Nonlinear model predictive control (NMPC) [32] is famous for its ability to improve local tracking precision. It performs periodic real-time optimization according to the prediction time window to achieve the purpose of iterative control to reduce tracking error. Through the numerical optimization algorithm proposed above, the global trajectory based on the kinematic and dynamic constraints of the USV can be obtained, in which the reference control quantity can be obtained. Therefore, the trajectory optimization uses the error index of control quantity as the optimization target. Setting the current time as $t_{j}$ and the prediction time window as $W_{n}$, the optimization problem in terms of NMPC can be formulated as

(8) $$\begin{array}{c} min\;\frac{1}{2}\sum_{i=t_{j}}^{t_{j}+W_{n}}[{\left(X_{i}-X_{i}^{ref}\right)}^{T}W_{mpcx}\left(X_{i}-X_{i}^{ref}\right)+{\left(\tau_{i}-\tau_{i}^{ref}\right)}^{T}W_{mpc\tau }\left(\tau_{i}-\tau_{i}^{ref}\right)\\ +{\left(\tau_{i}-\tau_{i-1}\right)}^{T}W_{mpcu}\left(\tau_{i}-\tau_{i-1}\right)], \end{array}$$

where the first term represents the error between the state variable and the reference state variable, which is mainly used to improve the accuracy of state tracking and maintenance in the process of real-time control. The second term represents the error between the control variable and the reference control variable. This term is used to meet the index of the lowest energy consumption. Although this problem has been considered in detail in the context of optimization objectives in global trajectory planning, secondary planning in local tracking control can achieve better results. The third term can improve the smoothness of input variables in actual control and meet the needs of practical application control. $W_{mpcx}=diag\left\{10,10,4,2,2,2\right\}$, $W_{mpc\tau }=diag\left\{2,0,2\right\}$ and $W_{mpcu}=diag\left\{4,0,4\right\}$ represent the positive definite, cost and weight matrices, respectively. And considering the control requirements of real-time operation and stability, we choose $W_{n}$ to be 30, 0.05 s as the sampling period, and the cycle of the NMPC algorithm call to be 0.1 s.

## 5. Experimental Analysis

In Section 5, we perform simulation experiments using the open source Otter USV simulator [33] within the ROS environment. The Otter USV simulator is a catamaran 2.0-m long, 1.08-m wide and 1.06-m high. When fully assembled, it weighs 65 kg, and has the ability to be disassembled into parts weighing less than 20 kg, such that a single operator can launch the Otter from a jetty, lake, beach or riverbank. A PX4 drone autopilot is used as the UAV, which is mounted with a monocular camera. The Otter USV is traveling within a 200 × 100 square meter area, with many blocks placed therein as obstacles. We set up several different obstacle terrains to test the crossing ability of the USV-UAV cooperative system.

### 5.1. Obstacle Recognition Ability

**Figure 4:**
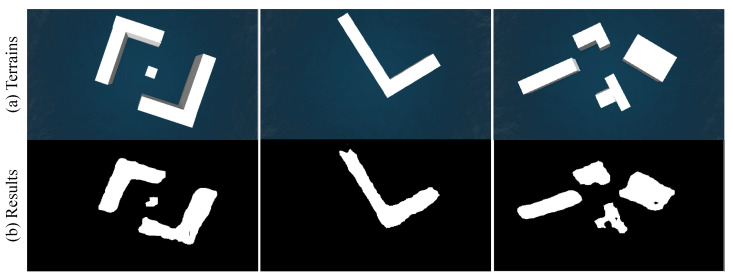
Obstacle recognition results of different terrains.

Firstly, we perform experiments on the ability of obstacle recognition by the USV monocular camera. The semantic segmentation algorithm is used to recognize objects. Several terrains are randomly placed in the virtual environment. Some of the segmentation results are shown in Figure 4, from which we can see that the proposed light-weight segmentation network can successfully identify obstacles in the environment. Although there are some empty areas in the middle or on the edges of the obstacles, the basic shape of the obstacles has been preserved. In the post-processing stage, image expansion can be used to increase the safe collision avoidance area and ensure the reliability of navigation. After that, 3D projection can be performed to convert the pixel information into 3D information in a global coordinate system.

### 5.2. Trajectory Generation Performance

**Figure 5:**
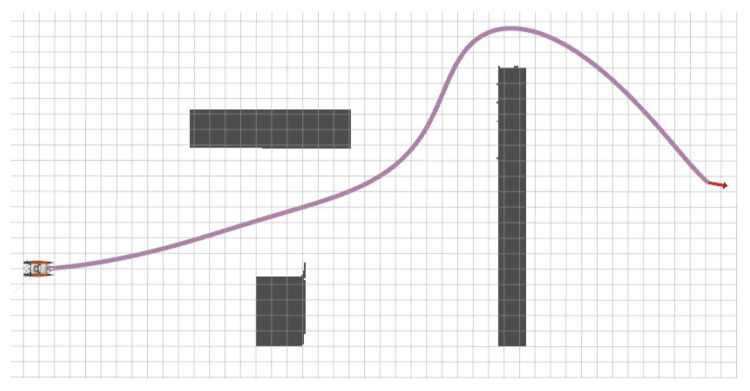
Global trajectory generation performance of the USV-UAV cooperative system.

The trajectory generation result is illustrated in Figure 5, from which we can see that the generated trajectory not only meets the collision avoidance condition, but also conforms to the hull’s kinematic characteristics. In the experiment, the Otter is an under-actuated USV and cannot provide direct lateral thrust during its operation. This requires that the running trajectory of the USV must be smooth enough, because too many bends will bring instability to the motion control of the USV and lead to the failure of path trajectory. The corresponding results can be seen in the subsequent path tracking control experiments.

**Figure 6:**
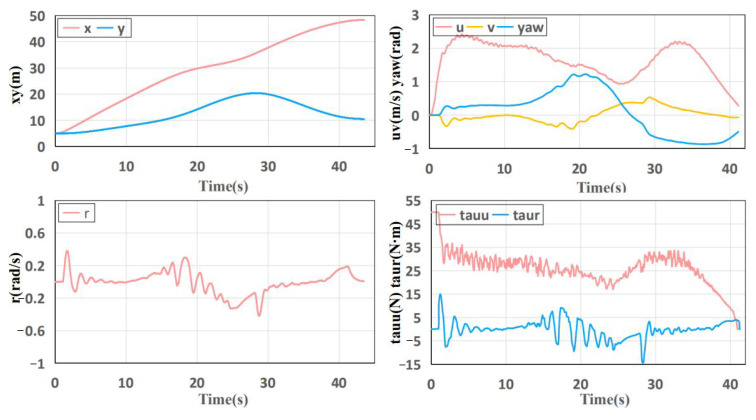
The changing trend of the state and control quantity of the USV with time.

The changing trend of the state and control quantity of the USV with time for the generated trajectory can be found in Figure 6. Overall, the quantities show a relatively gentle trend, especially for the *x* and *y* quantities, which verifies the smoothness of the trajectory. Higher order quantities such as *u*, *v* and yaw also present a gentle trend. Those are sufficient to show the effectiveness of the trajectory optimization method.

**Figure 7:**
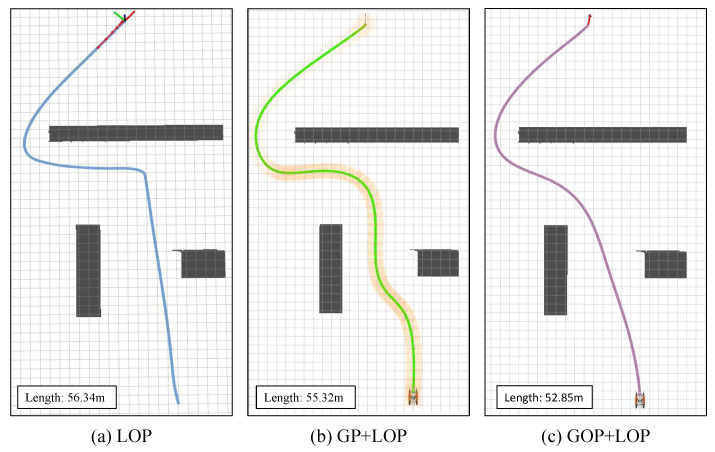
Trajectory generation comparison with different methods. LOP: trajectory generation with local optimization planning (global map provided by the UAV is unknown); GP+LOP: global planning without trajectory optimization; and GOP+LOP: the proposed method.

We also performed an ablation study on the proposed method. As shown in Figure 7, the LOP and GP+LOP methods are compared. LOP denotes the trajectory generation with local optimization planning, which means the global map provided by the UAV is unknown. Due to the limited perception field of the USV, it will take action to perform local trajectory planning unless it is near the obstacle. GP+LOP denotes global planning without trajectory optimization, which means the global map is known while trajectory optimization is not performed. Without the optimization stage, the generated trajectory shows a twisted shape, which is not optimal. GOP+LOP denotes the proposed method. In the lower left-corner of each sub-figure, the total length of the generated trajectory is shown. Our method obtains the shortest planned path with the best smoothness.

Here, we also compare the three methods quantitatively in Table 1. The indexes, such as RMSE, max error, speed and time, are evaluated by driving the hull to move. With the trajectory optimization method, the generated trajectory is more in line with the kinematic characteristics of the hull. As such, the tracking error, execution speed and control time achieve optimal values compared with other methods.

### 5.3. Tracking Control Performance

**Figure 8:**
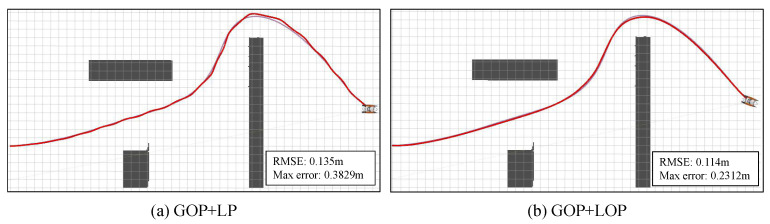
Tracking control performance comparison. GOP+LP denotes the tracking control method without optimization, i.e., the PID control. GOP+LOP denotes the proposed method with NMPC control.

To further verify the effectiveness of the proposed NMPC tracking control module, extensive comparative experiments are conducted. As shown in Figure 8, GOP+LP denotes the tracking control method without optimization, i.e., the plain PID with adjusted parameters. The proposed NMPC shows better tracking control performance qualitatively and quantitatively. There is no prediction time window for GOP+LP, so there will be many minor adjustments, resulting in an actual motion trajectory that is not smooth.

**Figure 9:**
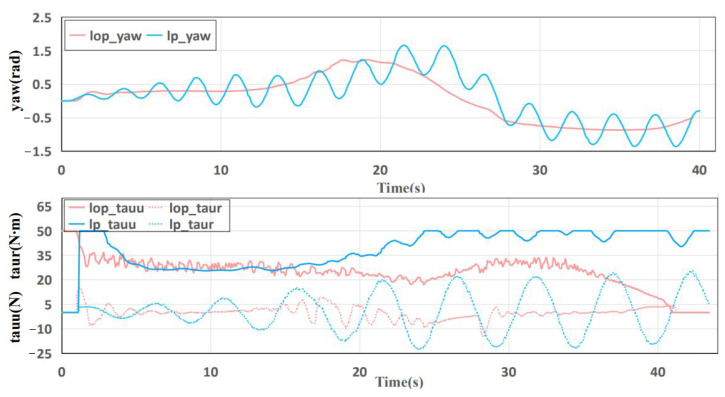
Execution state comparison of motion tracking control.

The execution states of different tracking control methods are visualized in Figure 9, from which the plain PID control shows unstable tracking states. Especially for the control input, the $\tau_{r}$ shows a divergent trend, which may lead to the input variable exceeding the controllable range and adversely affecting the motion control of the USV.

The quantitative comparison of tracking control methods can be found in Table 2, from which the proposed method shows better performance than GOP+LP (i.e., plain PID control). The proposed method not only achieves a smaller tracking control error, but also drives the USV at a quicker speed. Those particularly prove the effectiveness of the combination of motion control and trajectory generation with hull dynamics.

## 6. Conclusions

In this paper, a USV-UAV cooperative trajectory planning algorithm is proposed to overcome the problem of USV navigation in complex and multi-obstacle environments with an unknown global map. The proposed cooperative system is simple yet practical. In our method, the UAV acts as a flying sensor, providing a global map to the USV in real-time with semantic segmentation and 3D projection. Afterward, a graph search-based method is applied to generate an initial obstacle avoidance trajectory. An optimization method that considers the kinematic characteristics of the hull is proposed to make the trajectory more in line with the situation. Finally, an NMPC control method is applied to ensure high precision motion control of the USV. The proposed method has excellent performance and strong practicability in ocean engineering. In future work, we will verify the feasibility of the method in physical experiments and try to study the heterogeneous cooperation scheme of multi USV-UAV systems. 

## Figures and Tables

**Table 1:** Quantitative comparison of different trajectory generation methods.

Method	Length	RMSE	Max Error	Speed	Time
	(m)	(m)	(m)	(m/s)	(s)
LOP	56.34	0.120	0.3045	1.513	0.0667
GP+LOP	55.32	0.118	0.3047	1.608	0.0697
GOP+LOP	52.85	0.113	0.2312	1.675	0.0506

**Table 2:** Quantitative comparison of tracking control methods.

Method	RMSE	Max Error	Speed
	(m)	(m)	(m/s)
GOP+LP	0.135	0.3829	1.327
GOP+LOP	0.113	0.2312	1.675

## Data Availability

Data will be made available upon request from the authors.
